# Lipid Nanoparticles with Stiripentol and Cannabidiol Oil: From Rational Optimization to Preclinical Characterization

**DOI:** 10.3390/pharmaceutics18040503

**Published:** 2026-04-19

**Authors:** Sebastián Scioli-Montoto, Martin Lobos, Mauricio Melis, Santiago Ruatta, Giuliana Muraca, Cecilia Yamil Chain, Sebastián Cisneros, Vera Alejandra Alvarez, German Islan, Alan Talevi, María Esperanza Ruiz

**Affiliations:** 1 Laboratorio de Investigación y Desarrollo de Bioactivos (LIDeB), Facultad de Ciencias Exactas, Universidad Nacional de La Plata (UNLP), La Plata 1900, Argentina; scioli.montoto@biol.unlp.edu.ar (S.S.-M.);; 2 Consejo Nacional de Investigaciones Científicas y Técnicas (CONICET), CCT, La Plata 1900, Argentina; 3 Instituto de Investigaciones Fisicoquímicas Teóricas y Aplicadas (INIFTA—CONICET—UNLP), La Plata 1900, Argentina; 4 Instituto de Investigación en Ciencia y Tecnología de Materiales (INTEMA—CONICET—UNMdP), Mar del Plata 7600, Argentina; 5 Laboratorio de Nanobiomateriales, Centro de Investigación y Desarrollo de Fermentaciones Industriales (CINDEFI—CONICET—UNLP), Facultad de Ciencias Exactas, Universidad Nacional de La Plata, La Plata 1900, Argentina

**Keywords:** cannabidiol, Dravet syndrome, lipid nanoparticles, nanostructured lipid carriers, oral administration, pediatric formulations, pharmacokinetic study, release profile, response surface, stiripentol

## Abstract

**Background/Objectives**: Dravet Syndrome (DS) is a severe form of epilepsy that typically manifests in the first year of life and often requires polytherapy with two or more antiseizure medications (ASMs) to achieve adequate seizure control. Whereas the combination of stiripentol (STP) and cannabidiol (CBD) has demonstrated clinical efficacy, it presents significant formulation challenges due to the low aqueous solubility and poor oral bioavailability of both compounds. Furthermore, the high daily dosages of STP (approximately 50 mg/kg/day or higher) and the oily nature of conventional CBD formulations often hinder patient compliance, as pediatric patients frequently reject these treatments due to unfavorable organoleptic properties. **Methods**: Nanostructured lipid carriers (NLCs) containing STP and CBD suspended in an aqueous medium were developed. The formulation was optimized using Response Surface Methodology (RSM) and subjected to comprehensive in vitro and in vivo characterization. **Results**: The optimized formulation exhibited a mean particle size of 175.3 nm, a polydispersity index (PDI) of 0.232, a zeta potential of −8.35 mV, and an encapsulation efficiency greater than 99% for both drugs. Physicochemical characterization via atomic force microscopy, differential scanning calorimetry, thermogravimetric analysis, X-ray diffraction, and Fourier transform infrared spectroscopy revealed spherical nanoparticles without aggregation, with the drugs molecularly dispersed within the lipid matrix. Both STP and CBD showed sustained release profiles and demonstrated oral pharmacokinetic profiles that were comparable or superior to current commercial products. **Conclusions**: This novel formulation represents a promising therapeutic alternative for DS, enabling the co-administration of STP and CBD while potentially enhancing CBD bioavailability and treatment adherence in pediatric populations.

## 1. Introduction

Among epileptic encephalopathies, Dravet syndrome (DS) is one of the most severe and potentially life-threatening neurological disorders [1,2], characterized by early childhood onset, drug resistance, and significant neurodevelopmental impairment [3]. Despite advances in pharmacological therapies, DS treatment remains complex and often requires high doses of antiseizure medications (ASMs) and combination regimens to control severe, treatment-resistant seizures [4].

Among the currently approved treatments, stiripentol (STP) and cannabidiol (CBD) have demonstrated clinically significant reductions in seizure frequency when used in combination, improving patient outcomes [5]. STP ((E)-1-(1,3-benzodioxol-5-yl)-4,4-dimethylpent-1-en-3-ol) is a synthetically derived aryl allylic alcohol, belonging to a family of α-ethylene alcohols with activity in the central nervous system [6]. In contrast, CBD is a plant-derived cannabinoid of the terpenophenol class, structurally composed of a terpene moiety linked to a phenolic aromatic ring [7]. Both drugs exhibit distinct mechanisms of action; furthermore, pharmacokinetic studies in both healthy volunteers and patients indicate that CBD administration leads to a marginal increase in STP plasma concentrations, likely due to CBD-mediated inhibition of CYP2C19 [8].

However, while therapeutic efficacy has been established, formulation challenges remain largely unexplored. CBD is characterized by low aqueous solubility, documented at less than 0.01 mg/mL, and exhibits oral bioavailability values between 6% and 14% [9,10]. Similarly, STP is classified as a water-insoluble compound [11,12] and, although the absolute bioavailability of STP in humans remains undetermined due to the lack of an intravenous formulation for comparative assessment [13], research in rhesus monkeys has indicated a value of approximately 20% [12]. Given these characteristics and the fact that these formulations are intended for pediatric patients, STP and CBD are commonly administered as an oral suspension and a lipid solution (in sesame oil), respectively. These formulations are frequently associated with an unpleasant taste, an oily mouthfeel, and a lingering aftertaste, which can compromise treatment adherence, especially in young children requiring chronic, high-dose therapy [14,15,16,17]. It should be noted that, although a hard capsule formulation of STP is commercially available, it is generally unsuitable for infants and young children.

Poor palatability is a critical yet underestimated determinant of adherence in the management of pediatric conditions [18,19]. In chronic neurological disorders requiring lifelong treatment, even modest reductions in compliance can significantly impact seizure control, hospitalization rates, and overall quality of life [20]. Our laboratory provides a free therapeutic drug monitoring service for STP, CBD, Clobazam, and Norclobazam for children with DS in Argentina (https://lideb.biol.unlp.edu.ar/?page_id=97, (accessed on 13 April 2026)). Based on caregiver reports, the administration of a high-dose STP oral suspension and CBD oil is particularly challenging due to frequent rejection of these dosage forms by pediatric patients. In this sense, the organoleptic burden of currently available STP and CBD pharmaceutical products represents a relevant pharmaceutical and clinical limitation.

Lipid-based nanocarriers have emerged as promising platforms to enhance the delivery of poorly soluble drugs [21]. Among these nanosystems, nanostructured lipid carriers (NLCs) offer improved drug encapsulation, protection from degradation, controlled release, and the potential to modulate sensory perception by reducing direct contact between the active compound and taste receptors [22]. In pediatric pharmacotherapy, such systems may provide dual benefits: enhanced biopharmaceutical performance and improved palatability.

This study aims to evaluate the ability of NLCs to encapsulate both STP and CBD, enabling the co-administration of both drugs while potentially improving their pharmacokinetic properties and treatment adherence in pediatric populations by masking undesirable palatability-related characteristics.

## 2. Materials and Methods

### 2.1. Materials

Diacomit (Biocodex, Gentilly, France) was a gift from Francisco Vilavedra MD (pediatric neurologist at Dr. Noel Sbarra Children’s Hospital, La Plata, Argentina), and the CBD 100 mg/mL solution (Kanbis^®^) was obtained from the Elea Laboratory (Laboratorio Elea SACIFyA, Buenos Aires, Argentina). Reference standards of STP and benzyl paraben (BP) were purchased from the Cayman Chemical Company (Ann Arbor, MI, USA), and Sigma-Aldrich (Buenos Aires, Argentina), respectively. A 1 mg/mL standard solution of CBD in methanol was provided by the National Institute of Industrial Technology (INTI, Buenos Aires, Argentina).

The solid lipid myristyl myristate (Crodamol^®^ MM) and caprylic/capric triglyceride (Crodamol^®^ GTCC) were kindly donated by Croda Argentina (Tigre, Argentina). Poloxamer 188 (Kolliphor^®^ P188) and the cellulose dialysis tubing membrane were purchased from Sigma-Aldrich Argentina (Buenos Aires, Argentina).

HPLC-grade methanol and acetonitrile and analytical-grade KH_2_PO_4_ were purchased from Anedra (Research AG, Los Troncos del Talar, Argentina).

Other reagents and solvents used were HPLC or analytical grade.

### 2.2. Stiripentol Purification from Oral Suspension Powder

Diacomit^®^ powder was suspended in methanol, allowing the active pharmaceutical ingredient to be separated from insoluble excipients. Then, the suspension was filtered using Whatman filter paper (Buckinghamshire, UK) to eliminate insoluble particles. The remaining filtrate was mixed with silica powder (silica gel 60, Merck KGaA, Darmstadt, Germany) in a 1:2 ratio (commercial powder:silica powder). The mixture was then evaporated to dryness using a rotary evaporator (Büchi R200, Büchi, Flawil, Switzerland).

The dried powder was then loaded onto a column containing dichloromethane-packed silica gel 60, and the adsorbed STP was extracted using the same solvent. Finally, the extract was vacuum-dried to obtain purified STP crystals. Mean purity (by HPLC): 96.6% *w*/*w* (SD: 1.9).

### 2.3. Optimization of the Formulation by Response Surface Methodology Using a Central Composite Design

Statistical optimization of the particle size, zeta potential (Z-potential) and polydispersity index (PdI) of the nanosystem was carried out by Response Surface Methodology (RSM) using a Central Composite Design (CCD) applied to the two main factors that usually affect the final performance: the amount of solid lipid (X_1_, mg) and the amount of surfactant (X_2_, mg) [23]. The CCD is composed of three different groups of design points: two-level factorial design points, central points, and axial points [24]. Therefore, each factor selected for this optimization was evaluated at five different levels: −α, −1, 0, +1, and +α. To ensure the rotatability of the design, an α value of 1.412 was employed [25]. Real and coded values of the experimental design are presented in Table 1. The matrix generated consisted of thirteen different formulations, including four factorial points, four axial points and five center points (to allow a more reliable estimation of pure experimental error).

### 2.4. Preparation of Lipid Nanosystems

NLCs containing STP and CBD were prepared using the homogenization by ultrasonication technique, as reported in previous works [23,26,27]. Briefly, solid myristyl myristate (amount according to experimental design) is placed in a 10 mL beaker, which is then put in a thermostatic water bath set to 60 °C. After the solid lipid is melted, either an inert oil, Crodamol^®^ GTCC (50 µL), or a bioactive oil, Kanbis^®^ (50 µL, equivalent to 5 mg of CBD), is added for nanostructuring. At this point, 100 mg of STP is added to the oil phase until complete dissolution.

The aqueous phase was prepared as a poloxamer solution (according to the experimental design) and thermostated at the same temperature as the oily phase. Once both phases reached the same temperature, they were mixed and immediately ultrasonicated using a sonicator equipped with a 6 mm probe (130 W, Cole-Parmer, Antylia Scientific, Chicago, IL, USA) set to 80% of its power and run for about 15 min (corresponding to an energy input of 15 kJ). After the process was completed, the system was allowed to cool at room temperature.

### 2.5. STP and CBD HPLC-UV Quantification Method

Sample analysis was carried out by high-performance liquid chromatography (HPLC) using a Dionex Ultimate 3000 system equipped with a dual-gradient pump (DGP-3000) and a diode-array detector (DAD-3000) (Thermo Scientific, Sunnyvale, CA, USA). Separation was achieved on an ODS Hypersil C18 column (100 mm × 4.6 mm i.d., 5 μm; Thermo Fisher Scientific, Waltham, MA, USA). Elution was performed using a gradient system (see Table 2) composed of KH_2_PO_4_ buffer (10 mM, pH 7.0), methanol, and acetonitrile at a flow rate of 1.0 mL/min. UV detection was performed at 265 nm (STP) and 230 nm (CBD).

Samples from entrapment efficiency, drug loading capacity and release kinetics assays were injected directly or diluted with the mobile phase of the initial composition.

Pharmacokinetic study samples were prepared by the addition of 5.0 µL of a 600 mg/L BP solution in methanol (as internal standard) to 100 µL of mouse plasma. Then, 100 µL of acetonitrile was added for protein precipitation, and the mixture was vortex shacked for 10 s and centrifuged at 2300× *g* for 10 min. The supernatant was separated and extracted with 1.0 mL of chloroform. After 20 s of vortex shaking and centrifugation for 10 min at 1500× *g*, the organic layer was collected and evaporated to dryness under a N_2_ stream at 40 °C. The dried residue was then resuspended in 100 µL of the mobile phase.

In all cases, samples were filtered through nylon membranes (0.22 µm pore size) prior to injection (20 μL, fixed loop).

### 2.6. Measurement of Entrapment Efficiency (%EE) and Drug Loading Capacity (%DL)

The %EE and %DL of each formulation obtained according to the experimental optimization design were determined by quantifying the concentration of the free (non-encapsulated) drug in solution. Approximately 500 µL of each sample was transferred to Microcon^®^ centrifugal filter devices (Merk Millipore, Burlington, MA, USA) and centrifuged at 10,000× *g* for 10 min. The concentration of the free drug in the filtrate was subsequently quantified by the HPLC method described in the previous section.

The %EE and %DL were calculated using the following equations:

(1) $$\%EE=\frac{W_{0}-\left(C_{fd}\times V_{f}\right)}{W_{0}}\times 100$$

(2) $$\%DL=\frac{W_{0}-\left(C_{fd}\times V_{f}\right)}{\mathrm{Lipid mass}\;(mg)}\times 100$$

where *W*_0_ represents the initial mass of drug incorporated into the formulation (mg), *C_fd_* represents the free drug concentration in the filtrate (mg/mL), and *V_f_* corresponds to the final volume (mL) of the formulation, set to 10 mL.

### 2.7. Particle Size, Particle Distribution and Z-Potential

The mean particle size (hydrodynamic diameter, nm) and particle distribution (measured as PdI) of all formulations that were prepared were measured using a Nano ZS Zetasizer instrument (Malvern Instruments, Worcestershire, UK) and the dynamic light scattering (DLS) technique, using 1 mm path length polystyrene cuvettes. Z-potential, on the other hand, was measured using laser Doppler electrophoresis (LDE) using a Folded Capillary Zeta Cell (DTS1070) (Malvern Panalytical, Almelo, The Netherlands). All samples were diluted 1/100 with Milli-Q water (Merk Millipore, Burlington, MA, USA) and measured by triplicate at 25 °C.

### 2.8. Microscopic Analysis

Nanoparticles were evaluated by atomic force microscopy (AFM). A sample of the optimized formulation was diluted 1:1000 with ultrapure water (Milli-Q^®^, MA, USA) and placed onto a freshly cleaved mica surface, followed by the addition of 10 μL of a 10 mM CaCl_2_ solution. After 15 min, the plate was rinsed with ultrapure water, and excess liquid was removed using filter paper (Cytiva, Maidstone, UK).

Imaging was performed at room temperature using a Multimode Nanoscope V system (Bruker Corporation, Santa Barbara, CA, USA) at the Nanoscopy Laboratory of the Institute for Theoretical and Applied Physical Chemistry Research (INIFTA, CONICET, La Plata, Argentina). Measurements were performed in tapping mode with an etched silicon RTesp probe (Bruker Corporation, Santa Barbara, CA, USA), characterized by a resonance frequency of 300 kHz, a force constant of 42 N/m, and a tip radius of 8–12 nm. Typical scan rates were set to 1 Hz.

The optimized sample was further evaluated by transmission electron microscopy (TEM) at the Electron Microscopy Service of CONICET Bahía Blanca (Buenos Aires, Argentina), using a JEOL 100 CX II microscope (JEOL, Tokyo, Japan) equipped with a Erlangshen ES1000W CCD camera (Gatan Inc., Pleasanton, CA, USA). Samples were diluted 1:100 with ultrapure water (Milli-Q), and ten microliters of the dilution was placed onto a Cu grid. Subsequently, a drop of phosphotungstic acid was added as a contrast-enhancing agent, the excess was removed using filter paper, and the grid was allowed to dry at room temperature.

### 2.9. Thermal Analysis

#### 2.9.1. Differential Scanning Calorimetry Analysis

Thermal analysis of the pure components and the nanoparticles was performed by Differential Scanning Calorimetry (DSC) (Pyris 1 DSC, PerkinElmer INC., Waltham, MA, USA). Assayed formulations included the optimized formulation (NLC STP/CBD) and a control of empty nanoparticles (NLC-vehicle). Both the raw material and formulations were frozen at −80 °C and subsequently freeze-dried using a Rificor L-A-B4 lyophilizer (Buenos Aires, Argentina) prior to the analysis. Each measurement was carried out under a nitrogen atmosphere at a heating rate of 20 °C/min, in the range of −50 to 400 °C, using standard aluminum sample cells with 4.0 to 23.0 mg of lyophilized samples.

For analyzing possible changes in the crystallinity index (CI%) of myristyl myristate wax after the nanoparticle preparation process, relative to the raw material, the following expression was used, according to previous works [23,27]:

(3) $$CI\left(\%\right)=\frac{{∆H}_{\mathrm{NLC dispersion}}\left[J\times g^{-1}\right]}{{∆H}_{MM}\left[J\times g^{-1}\right]\times C_{\mathrm{lipid phase}}\left[\%\right]}\times 100$$

where ${∆H}_{\mathrm{NLC dispersion}}$ represents the melting heat of myristyl myristate in the nanosuspension, ${∆H}_{MM}$ corresponds to the melting heat of pure myristyl myristate, and $C_{\mathrm{lipid phase}}$ represents the percentage lipid concentration in each formulation. For all calculations, a ${∆H}_{MM}$ of 234.5 J/g and a $C_{\mathrm{lipid phase}}$ of 2.68% were used. The ${∆H}_{\mathrm{NLC dispersion}}$ was calculated considering the melting peak corresponding to myristyl myristate. The heat flow signal (mW) was normalized by the sample mass to obtain mW/mg. The change of enthalpy was then determined by integrating the area under the endothermic peak. Since a constant heating rate was applied, the integration over time (in seconds) was equivalent to integration over temperature. The resulting values are expressed in mJ/mg.

#### 2.9.2. Thermogravimetric Analysis (TGA)

Thermal stability of the lyophilized formulations (NLC STP/CBD and NLC-vehicle) and raw materials was assessed by TGA using a TGA Q500 apparatus (TA Instruments, New Castle, DE, USA). Approximately 10 to 27 mg of each lyophilized sample was accurately weighed in platinum pans and analyzed at a heating rate of 10.00 °C/min under a 10 mL/min flow-rate nitrogen atmosphere.

### 2.10. Spectroscopic Analysis

#### 2.10.1. X-Ray Diffraction Analysis (XRD)

For the analysis of the crystalline structure of the optimized formulation, empty nanoparticles and raw materials, a PANalytical X’Pert PRO diffractometer equipped with a CuKα X-ray source operating at 40 kV and 40 mA (Philips PW 1830, PANalytical, Almelo, The Netherlands) was employed. Diffraction spectra of the freeze-dried samples were recorded over a 2θ range of 5–60° using a step size of 0.02° and an acquisition time of 1 s per step.

#### 2.10.2. Fourier Transform Infrared Spectroscopy

The behavior of the different components of the formulation after the synthesis process and the presence of possible interactions between them were studied by Fourier Transformed Infrared Spectroscopy (FTIR) using a JASCO FT/IR–4200 spectrometer, with 256 scans for background correction, a high-energy ceramic source, and a DLARGS detector (Jasco Inc., Easton, MD, USA). Freeze-dried samples were prepared in a solid state with 5% (*w*/*w*) KBr.

### 2.11. In Vitro Release Assay

The release behavior of STP and CBD from the NLC STP/CBD formulation was evaluated in a medium containing 50 mM KH_2_PO_4_ (pH 6.8) with 0.5% *w*/*v* sodium dodecyl sulfate (SDS), in triplicate. Briefly, 0.5 mL of the optimized formulation was added to 9.5 mL of the release medium in a 50 mL beaker and immediately placed in a heated orbital shaker (model BM081, Biomint, Buenos Aires, Argentina) set to 37 °C and 100 rpm. As a control, the release of both drugs from their commercial products, Diacomit^®^ and Kanbis^®^, was evaluated under the same conditions. Samples of 0.5 mL were withdrawn at predefined time intervals (from 0.5 to 8 h), and the extracted volume was replaced with fresh medium kept at the same temperature. After collection, all samples were double-filtered using an in-house device equipped with nylon membranes of 0.22 µm pore size and assisted by centrifugation at 6000× *g* for 3 min. Finally, STP and CBD in solution were quantified by the HPLC methodology previously described.

### 2.12. Pharmacokinetic Study

A total of 54 male BALB/c mice, specific-pathogen-free, 6–8 weeks old and weighing 22–26 g, were provided by the Faculty of Veterinary Sciences, National University of La Plata (FCV-UNLP). Animals were housed in groups of four per cage under controlled temperature conditions and a 12 h light/dark cycle, with environmental enrichment and *ad libitum* food and water. Upon arrival, mice were allowed to acclimatize for a minimum of seven days before any experimental procedure, followed by five days of adaptation to the oral gavage. All experiments were performed during the light phase of the cycle.

In addition to the optimized NLC STP/CBD formulation, commercial products of STP (Diacomit^®^) and CBD (Kanbis^®^) were evaluated for comparison purposes. Samples were administered as a single dose by oral gavage at a volume of 20 mL/kg using a 20 gauge curved gavage needle attached to a 1 mL syringe. Blood samples were then collected at 0.5, 1, 2, 4, 8 and 24 h post-administration (h post-dose). Three mice were used for each time point.

Blood samples were collected by intracardiac puncture immediately after euthanasia by carbon dioxide (CO_2_) inhalation and placed into microtubes containing EDTA as an anticoagulant. Then, plasma was separated by centrifugation at 1500× *g* for 10 min, and samples were prepared according to the method described earlier.

All experimental procedures involving animals were conducted in accordance with institutional guidelines and were approved by the Institutional Committee for the Care and Use of Laboratory Animals (CICUAL) of the Faculty of Exact Sciences, National University of La Plata (UNLP), under protocol number 018-06-15 (Supplementary Materials S1 and S2).

The sanitary health certificate of the animals used in this study is included in the Supplementary Materials S3.

### 2.13. Statistical Analysis

In the RSM optimization stage, the matrix of the CCD design was constructed, randomized and analyzed under R language (version 4.4.3) [28], using the *rsm* package (version 2.10.5) [29] and RStudio (version 2024.09.1+394) as the integrated development environment [30]. For final optimization, the *desirability* package (version 2.1) was used [31].

Statistical differences between groups in the pharmacokinetic study were evaluated using Student’s *t*-test for two-group comparisons. Before conducting this parametric test, the normal distribution and the homogeneity of variances were verified using the Shapiro–Wilks test and the F-test, respectively.

In all cases, statistical significance was established at *p* < 0.05.

## 3. Results and Discussion

### 3.1. Optimization Using RSM with a CCD

For the optimization of the nanosystem, a two-factor, five-level CCD was used. A 13-run experimental matrix was generated using the *rsm* package, and the mean particle size, PdI, and Z-potential were measured for each sample. Table 3 presents the CCD matrix and the obtained result for the three independent variables considered, expressed as mean ± standard deviation of three analytical replicates.

**Figure 1:**
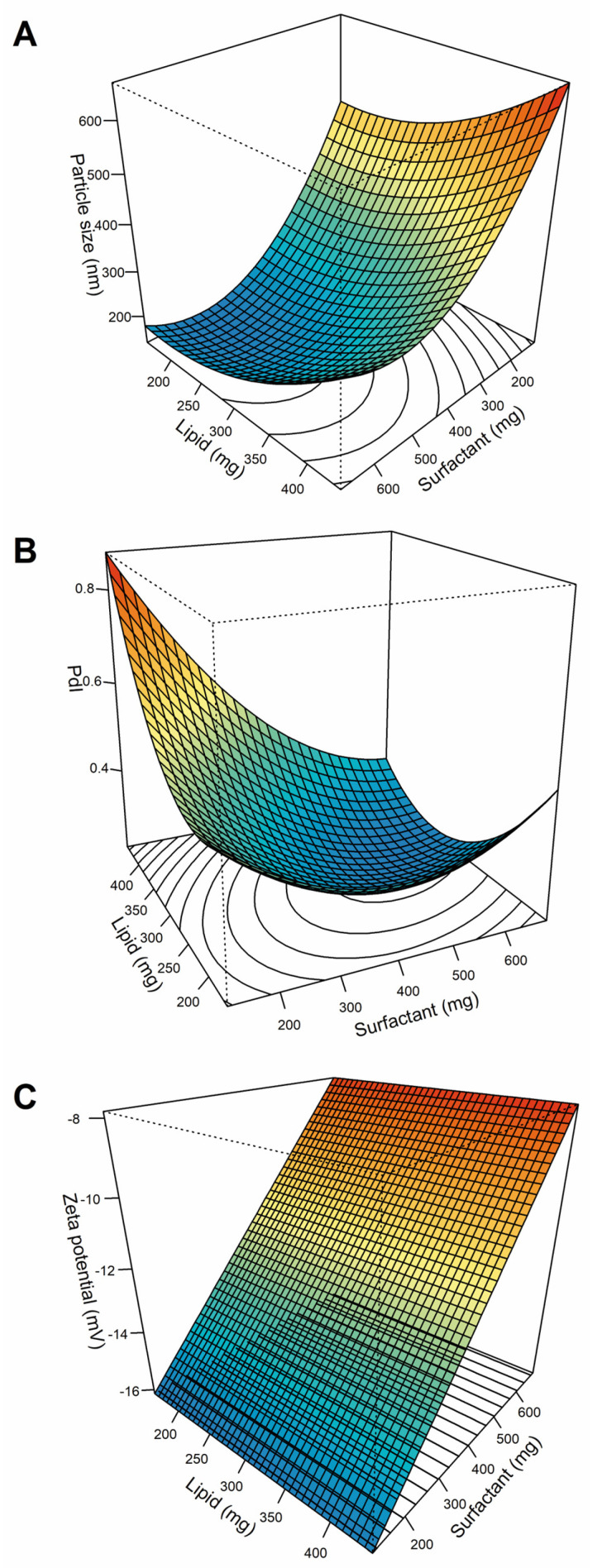
Response surface plots for (**A**) particle size; (**B**) polydispersity index (PdI); and (**C**) Z-potential.

After data analysis, particle size was best fitted by a pure quadratic model (*Y*_1_ = 798.9 − 1.135 × *X*_1_ − 2.027 × *X*_2_ + 0.00298 × *X*_1_^2^ + 0.001862 × *X*_2_^2^; *p* < 0.05), PdI by a second-order model (*Y*_2_ = 0.9387 − 0.00318 × *X*_1_ − 0.00078 × *X*_2_ − 3.40 × 10^−6^ × *X*_1_*X*_2_ + 8.14 × 10^−6^ × *X*_1_^2^ + 1.62 × 10^−6^ × *X*_2_^2^; *p* < 0.05), and Z-potential by a linear model (*Y*_3_ = −17.87 + 0.01467 × *X*^2^; *p* < 0.01). The response surface plots for the different responses are shown in Figure 1.

Following model fitting to the experimental data obtained from the design, formulation optimization was performed using a desirability function approach. A desirability value of 0.822 was obtained for an amount of lipid of 268.4 mg and surfactant of 553.4 mg. For these selected levels, the model predicted a particle size of 157.5 nm, a PdI of 0.231, and a Z-potential of −9.75 mV.

After synthesizing the optimized formulation according to the selected conditions, the experimentally measured values (with their corresponding relative prediction error, %) were 175.3 nm (10.2%), 0.232 (0.43%), and −8.35 mV (16.8%), for particle size, PdI, and Z-potential, respectively.

The optimized formulation reached encapsulation efficiencies (%EE) above 99% for both STP and CBD, which is consistent with the high lipophilicity of both drugs. On the other hand, drug loading (%DL) was 31.8% for STP and 1.6% for CBD.

### 3.2. Physicochemical and Morphological Characterization

To complement the DLS measurements of the optimized formulation, AFM was employed. In the case of lipid nanoparticles, AFM was used to provide qualitative information on particle morphology, as particle deformation during sample deposition and drying typically occurs with this technique, limiting the accurate estimation of the true dimensions [32].

**Figure 2:**
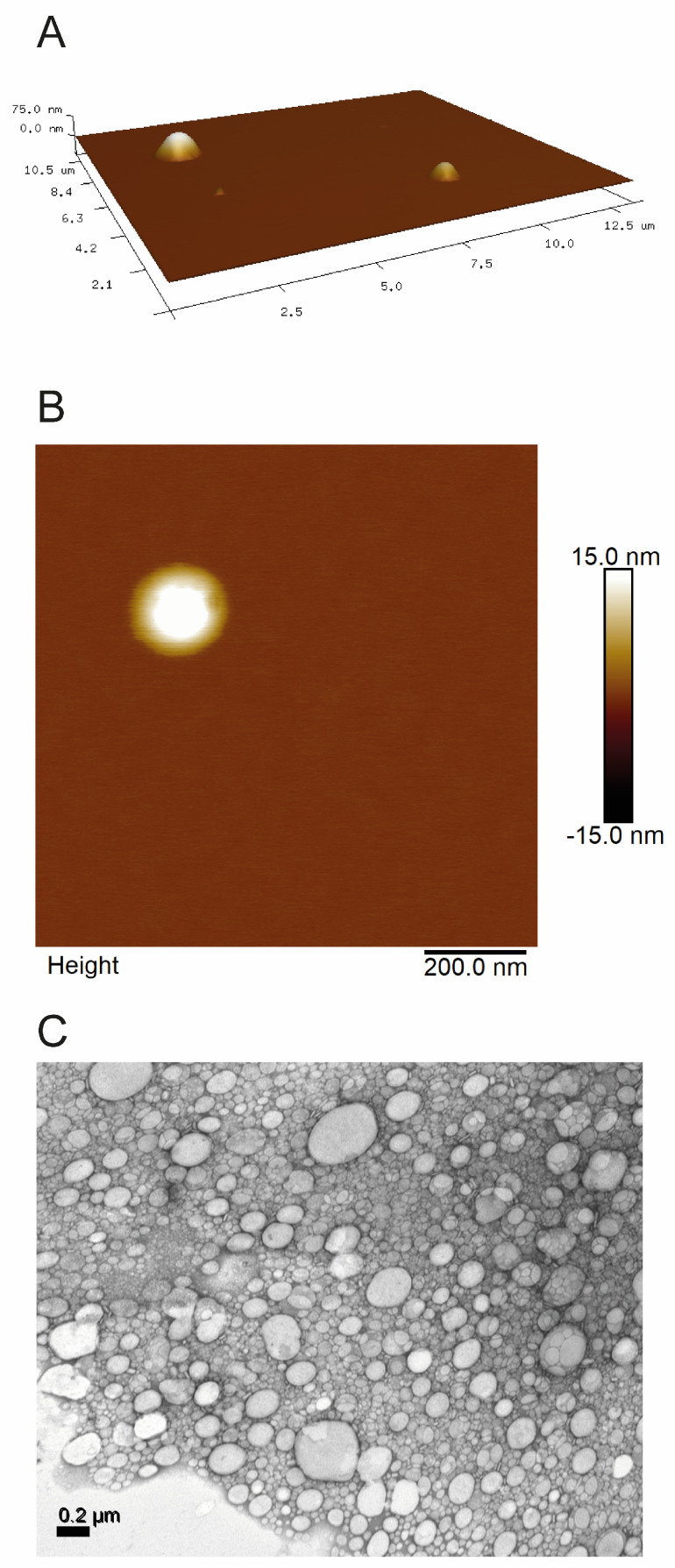
(**A**) 3D AFM image of NLC STP/CBD; (**B**) 2D AFM image of an isolated nanoparticle, and (**C**) TEM image of NLC STP/CBD.

Figure 2 presents 3D (A) and 2D (B) images of the formulation, showing nanoparticles with spherical morphology and no aggregation. Similar to previous reports [23,26], particle deformation due to sample preparation, as well as interaction with the probe, tends to produce higher diameter values (in the *xy* plane) and lower height values (in the *z* direction) than those determined by DLS.

As a second methodology for morphology evaluation, and to confirm the results obtained by AFM, TEM was also employed. Figure 2C shows an image obtained by the latter technique, confirming the spherical morphology of the nanoparticles, with a mean particle size of 160.8 ± 28 nm, calculated using software ImageJ (version 1.54g) [33].

It is important to highlight that size characteristics, as well as Z-potential and PpI, remained stable for a three-month period. No significant differences were found (*p* > 0.05) between measurements of these three variables (*N* = 4) between 0 and 90 days post-production, for a batch stored under refrigeration (2–8 °C).

**Figure 3:**
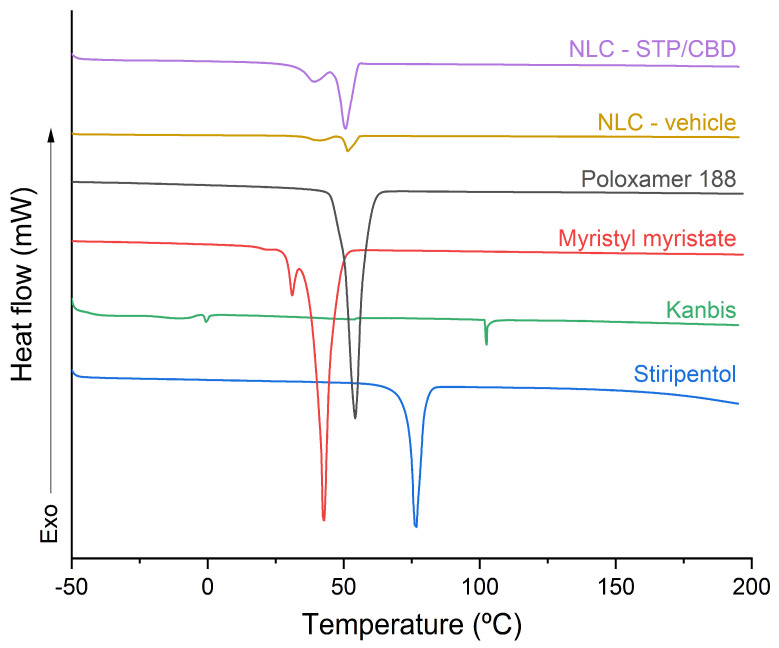
Stacked DSC thermograms of raw materials (STP, Kanbis, myristyl myristate and poloxamer 188), NLC-vehicle and NLC STP/CBD.

Raw materials, the NLC-vehicle and NLC STP/CBD were thermally evaluated by DSC and TGA, and the corresponding thermograms are shown in Figure 3. As for STP, its thermogram shows an endothermic peak at around 76.5 °C with a melting heat of 28.88 kJ/mol, corresponding to the reported melting point of STP [34].

In the case of Kanbis, the peak corresponding to the melting process of CBD (around 66 °C) [9,35,36] was not observed, which is to be expected for a formulation in which the drug is dissolved in sesame oil and therefore is not present in its crystalline state. Two small endothermic peaks can be observed at approximately 0 and 100 °C, which could be related to phase transitions of the residual water present in the sample [37].

The thermogram corresponding to myristyl myristate shows two endothermic peaks at 31 and 42.6 °C. The first one could be associated with the α polymorph, characterized by a lower lattice energy and lower melting point, and the second one could correspond to the β’ polymorph, which presents higher lattice energy [38]. For poloxamer 188, on the other hand, only one endothermic peak is observed at around 54 °C, corresponding to its melting point [23,39].

Regarding the NLC-vehicle and NLC STP/CBD, two main endothermic peaks can be observed: the first at approximately 41 °C, which could correspond to the melting process of myristyl myristate in the β’polymorphic form present in the nanoparticles, and the second at 51 °C, which could correspond to the melting process of poloxamer 188.

The absence of the endothermic peaks associated with the melting process of STP suggests that this active pharmaceutical ingredient does not rearrange into crystalline structures after the synthesis process; rather, it remains molecularly dispersed or in an amorphous state within the lipid matrix. For CBD, however, the absence of a characteristic melting peak could be also related to the detection limit of the DSC equipment. At such low concentrations (approximately 0.5% *w*/*w* of the lyophilized mass), the thermal signal of the drug may be masked by the dominant signals of the lipid matrix and surfactant. However, it also should be noted that CBD is incorporated into the formulation as a solution (Kanbis); therefore, the presence of crystalline CBD is not expected in original, non-lyophilized samples.

Furthermore, the results obtained from the calculation of the crystallinity indices were consistent with the previously stated hypothesis, yielding values of 9.12% for the NLC-vehicle and 7.16% for NLC-STP/CBD. These findings suggest a decreased crystalline arrangement in the NLC lipid matrix relative to the raw material [40].

**Figure 4:**
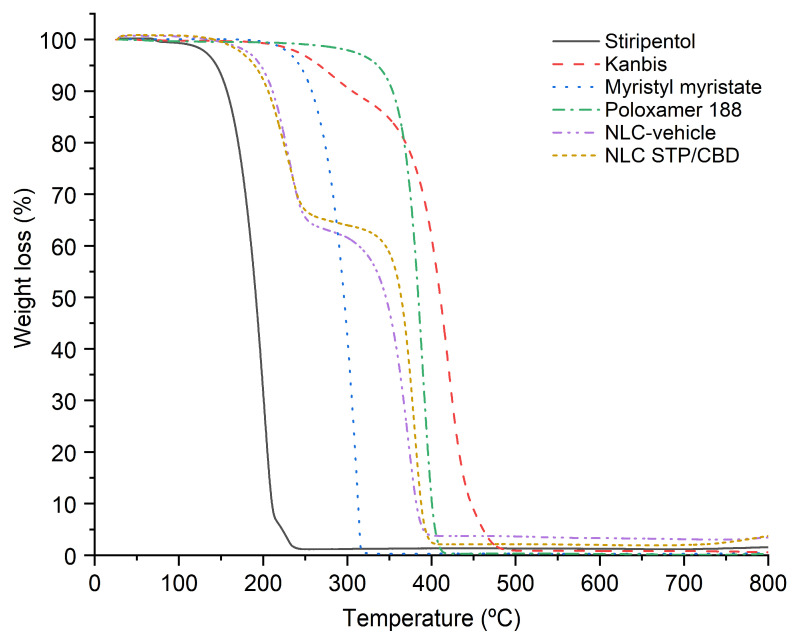
TGA thermograms of, STP, Kanbis, myristyl myristate, poloxamer 188, NLC-vehicle and NLC STP/CBD, obtained in the range of 25–800 °C.

TGA was used to evaluate the thermal stability of the formulation components under the temperature conditions reached during nanoparticle obtention, where temperatures can reach values of up to 90 °C. As can be seen in Figure 4, all the components of the formulation were stable under the preparation conditions, as no mass losses were observed below 100 °C. STP showed a single-step mass-loss event, between 114 and 250 °C, corresponding to its thermal decomposition. On the other hand, Kanbis presented a first degradation process in the range of 220 and 320 °C (accounting for a 10% loss of the sample weight), attributable to the CBD present in the product [36,41], and a second process between 300 and 500 °C, corresponding to sesame oil [42], the main component of the pharmaceutical product. Thermal decomposition of myristyl myristate occurred in a single-step process between 150 and 330 °C, with a mass loss of approximately 100%, whereas poloxamer 188 showed a single weight-loss step between 281 and 420 °C, in agreement with our previous work [23]. Both the NLC-vehicle and NLC STP/CBD exhibited a two-step degradation process. The first one was between 120 and 260 °C, with a mass loss of 35%, and the second one was in the range of 260–410 °C, with a mass loss of 65%. Since the thermogram of NLC STP/CBD was practically identical to that of the NLC-vehicle, none of the thermal processes observed for NLC STP/CBD can be attributed to the decomposition of the incorporated active ingredients. Instead, they are more likely associated with the main components of the formulation, myristyl myristate and poloxamer 188, whose ratio in the optimized formulation is 33 to 67, respectively.

These findings support the results obtained by DSC, providing additional evidence that the drugs are molecularly dispersed within the lipid matrix rather than present as crystalline domains.

**Figure 5:**
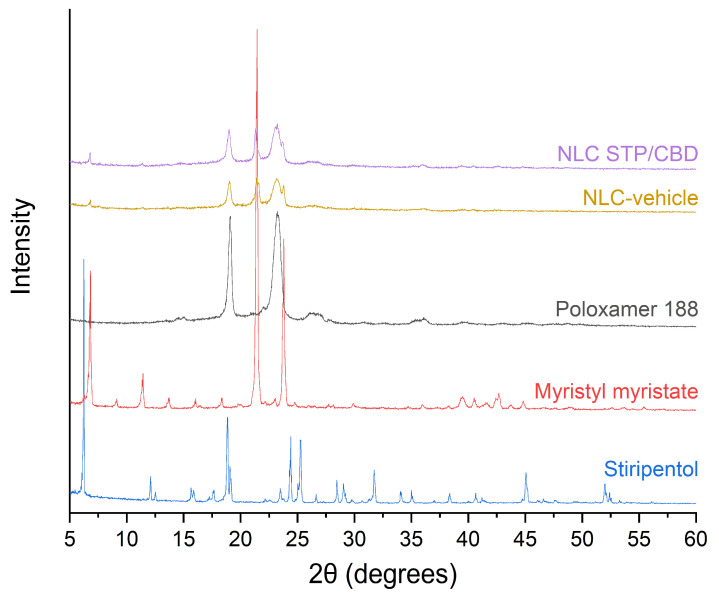
XRD diffraction patterns corresponding to the main components of the formulation, NLC-vehicle and NLC STP/CBD.

Figure 5 shows the XRD diffraction patterns of the main components of the formulation, as well as the NLC-vehicle and the optimized formulation. For STP, the main Braggs reflections were observed at (2θ) angles of 6.05°, 12.11°, 15.65°, 15.93°, 17.65°, 18.83°, 19.10°, 24.41° and 25.29°, in agreement with previous works [34,43]. In the case of myristyl myristate, the diffraction pattern exhibited the characteristic Bragg reflections at 21.41° and 23.80°, corresponding to d-spacing values of 0.41 nm and 0.37 nm, respectively, which can be attributed to the β’ polymorph [44]. Poloxamer 188, on the other hand, showed two main peaks at 19.08° and 23.26°, with d-spacing values of 0.46 nm and 0.38 nm, respectively, consistent with previous reports [23,45]. Diffraction patterns of the NLC-vehicle and NLC STP/CBD displayed the characteristic peaks of individual excipients, resembling a physical mixture. The characteristic peaks of STP were not present, nor were the peaks reported in the literature for pure CBD [35,46], indicating the absence of these compounds in a crystalline form. Consistent with our previous discussion, the existence of traces of crystalline CBD within the freeze-dried samples cannot be excluded, as these may fall below the sensitivity threshold of XRD analysis. These findings further support the results obtained from the DSC and TGA analyses.

The incorporation of the drug into the lipid matrix in a molecularly dispersed or amorphous state, as suggested by DSC and XRD analyses, allows not only a more homogeneous distribution of the drug along the formulation but also reduces the likelihood of nucleation and crystal growth due to restricted molecular mobility [47] and contributes to enhanced physical stability, reducing the probability of phase separation or drug expulsion [48].

**Figure 6:**
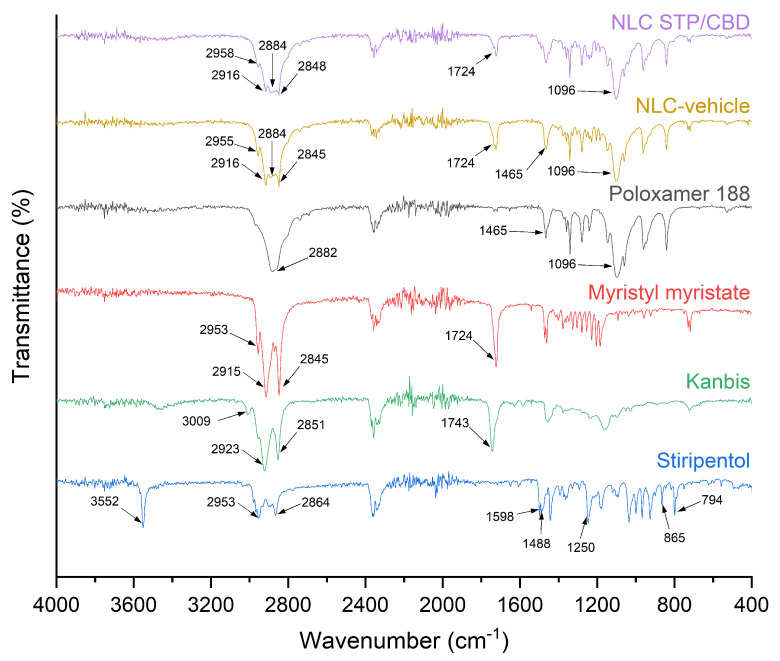
Overlaid FTIR spectra of STP, myristyl myristate, poloxamer 188, NLC-vehicle and NLC STP/CBD.

Possible interactions among the different components that are part of the formulation were analyzed by FTIR. The stacked IR spectra of the raw components, the NLC-vehicle, and NLC STP/CBD are shown in Figure 6. For pure STP, absorption bands characteristic of its molecular structure can be seen [49]: a sharp band at 3552 cm^−1^, corresponding to the O-H functional group, and bands at 2953 and 2864 cm^−1^, which can be attributed to the stretching vibrations of aliphatic C-H groups, present in H-C=C-H and C-H, respectively. Bands at 1598 and 1488 cm^−1^ can be assigned to stretching vibration of the C=C bonds of the aromatic ring. On the other hand, the band at 1250 cm^−1^ could be attributed to C-O-C stretching vibrations of the methylenedioxy group (-O-CH_2_-O-), confirming the presence of the 3,4-methylendioxyphenyl moiety. Finally, absorption at 865 and 794 cm^−1^, could be assigned to the out-of-plane C-H bending vibrations of the aromatic ring. Bands at 2360 and 2330 cm^−1^ are not consistent with vibrations of the STP molecule and are likely due to the asymmetric stretching of atmospheric CO_2_, appearing as artifacts in the FTIR spectra [50].

Kanbis, on the other hand, exhibits characteristic vibrational bands of sesame oil, the major component of the product [51]. Sesame oil is mainly composed of linolenic and oleic acids [52]; therefore, the absorption bands correspond to the main functional groups of these compounds. Peaks at 3009, 2923, and 2851 cm^−1^ can be attributed to the C-H stretching of cis double bonds (=C-H) and to symmetric and asymmetric stretching of aliphatic CH_2_ groups, while the peak at 1743 cm^−1^ corresponds to stretching of the ester carbonyl group present in triglycerides [53].

As in previous reports, myristyl myristate showed characteristic bands at 2953, 2915, 2845 and 1724 cm^−1^ that could be associated with the asymmetric stretching of C-H in -CH_3_ groups, the symmetric and asymmetric stretching of the C-H bond in -CH_2_- groups [54], and the stretching vibration of the C-O bond of the C=O group [55].

Poloxamer 188 showed bands at 2882 and 1465 cm^−1^ assignable to the symmetric stretching of C-H bonds present in the aliphatic chains of the copolymer and the bending vibration of -CH_2_- groups. Finally, the band at 1096 cm^−1^ can be associated with the symmetric stretching of C-O-C bonds [23].

For the NLC-vehicle and NLC-STP/CBD, the spectra showed vibrational bands corresponding to the main components, myristyl myristate and poloxamer 188, similar to what would be expected for a physical mixture of both excipients, without the appearance of the new bands or significant shifts indicative of strong chemical interactions.

### 3.3. In Vitro Release Assay

The release profiles of STP and CBD from NLC in phosphate buffer containing 0.5% *w*/*v* SDS exhibited distinctive kinetic behavior, which could be related to differential drug–matrix interactions and partitioning dynamics. In the release medium, SDS was used at concentrations above its critical micellar concentration (CMC; approximately 8 mM [56], equivalent to 0.23% *w*/*v*). This allows micelle formation and promotes sink conditions, enhancing the solubilization of highly lipophilic drugs and sustaining the concentration gradient necessary for diffusion. Under these conditions, the drug release process can be regarded as a micellar solubilization, i.e., not limited by aqueous solubility but rather controlled by diffusion from the lipid core toward the particle–medium interface and subsequent partitioning into SDS micelles [57].

**Figure 7:**
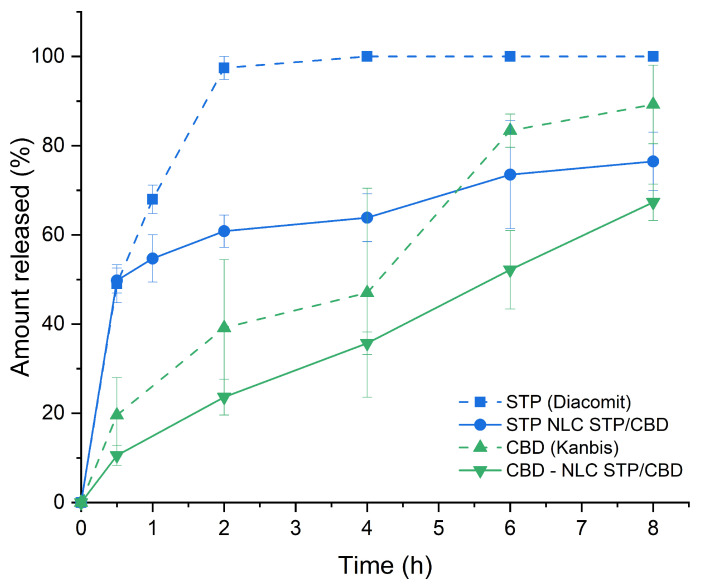
Release profiles of STP and CBD from NLC STP/CBD and commercial products in phosphate buffer 50 mM, pH 6.8, with 0.5% SDS. Error bars correspond to the standard error of the mean (SEM).

As can be seen in Figure 7, while the commercial powder of STP showed a typical immediate release oral solid dosage form profile (achieving 100% release in the first 2 h), STP from the NLC STP/CBD exhibited a marked initial burst release during the first 30 min, followed by a gradual release, reaching nearly 100% at 8 h. This biphasic pattern suggests that a fraction of STP may be localized at or near the surface of the nanoparticles or in a less ordered lipid domain [35]. In contrast, CBD displayed a slower and more sustained release profile without a pronounced burst, indicating a stronger integration with the lipid components of NLCs, consistent with previous reports [58]. The different release behaviors observed for STP and CBD could therefore be associated with differences in their diffusion rates among the lipid matrix and their partition coefficients between the nanoparticles and the micelles in the surrounding medium: STP has a LogP (the logarithmic form of the oil/water partition coefficient) value of 2.94 [59], while values for CBD have been reported from 6.5 to 7.5 [9,60].

### 3.4. Pharmacokinetic Study

**Figure 8:**
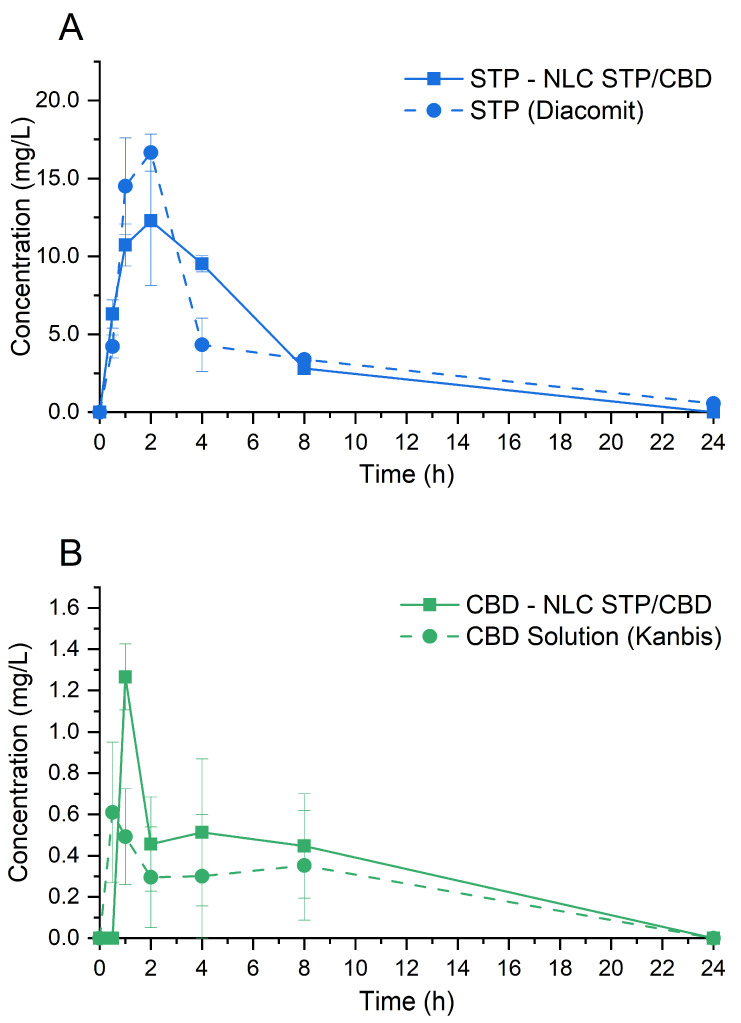
Pharmacokinetic profiles of STP (**A**) and CBD (**B**) from NLC STP/CBD and their commercial products (Diacomit^®^ and Kanbis^®^, respectively). Error bars correspond to the SEM.

The PK profiles obtained after oral administration of STP and CBD from NLC STP/CBD and their corresponding commercial products are shown in Figure 8. As can be seen, both drugs displayed similar plasma concentration vs. time curves, characterized by a rapid absorption of the active pharmaceutical ingredients shortly after administration, followed by a decline over the 24 h observation period, as typically observed after extravascular drug administration. Table 4 summarizes the values of maximum concentration (Cmax), time to reach maximum concentration (Tmax), and area under the concentration vs. time curve, from 0 to 24 h (AUC_0–24_), obtained after each administration.

When compared over time, the differences observed in plasma concentrations of STP and CBD obtained from the optimized formulation and the commercial product were not statistically different in any case (*p* > 0.05). However, the results in Table 4 and Figure 8 suggest a trend toward greater CBD bioavailability for NLC STP/CBD compared to the commercial product, as the former showed higher values of Cmax, Tmax (approximately double in both cases), and AUC_0–24_ (48% higher).

## 4. Conclusions

The pharmacological therapy of chronic diseases poses a persistent challenge, as it necessitates long-term patient adherence, sometimes extending throughout the patient’s lifetime. When it comes to a severe neurological disease affecting the pediatric population, achieving treatment adherence becomes even more demanding.

Hence, we aimed to develop a formulation based on NLCs loaded with STP and CBD, two drugs commonly used in combination for the treatment of DS, to enhance patient adherence by simplifying the dosing regimen and improving organoleptic characteristics, as it is odorless and virtually tasteless. Additionally, being a liquid product, it is compatible with enteral feeding, which is sometimes required for patients with DS. As detailed in the preceding sections, the formulation exhibited favorable physicochemical characteristics, a more sustained release profile compared to commercially available products, and comparable PK profiles, with a trend toward enhanced bioavailability for CBD.

A limitation of our development is, however, that combining drugs into a single product reduces dosage versatility, as the required dose ratio for both drugs may not remain constant throughout the course of treatment. The drug loading ratio of 20:1 (STP:CBD) of the optimized formulation corresponds to an established STP treatment regimen (at a dose of 50 mg/kg/day, for example), in which CBD co-administration is initiated at a dose of 2.5 mg/kg/day [61]. However, while this ratio may reflect the clinical starting point, maintenance CBD dosages may be adjusted up to 20 mg/kg/day based on individual response [62].

To address this issue, as well as to enable the administration of higher doses (e.g., for older children), we intend to optimize the lyophilization process of the NLCs, both for the combined drugs and for those encapsulated individually.

In summary, the results obtained so far represent a promising starting point for achieving a formulation capable of improving the treatment of children with DS by increasing treatment compliance through improved palatability and co-administration in a single dosage form.

## Figures and Tables

**Table 1:** Factors (X) levels and optimization objectives pursued for the three dependent variables (Y) of the CCD.

Factors	Levels				
	−α	−1	0	1	α
X_1_: Amount of lipid (mg)	158.6	200	300	400	441.4
X_2_: Amount of surfactant (mg)	117.2	200	400	600	682.8
Dependent variables	Objective				
Y_1_: particle size (nm)	Minimize				
Y_2_: Polydispersity index	Minimize				
Y_3_: Z-potential (mV)	Minimize				

**Table 2:** Gradient used for STP and CBD quantification. A: 10 mM KH_2_PO_4_, pH 7.0; B: methanol; C: acetonitrile.

Time (min)	% A	% B	% C
0	41	54	5
13	41	54	5
15	22	73	5
20	22	73	5
22	41	54	5
25	41	54	5
30	41	54	5

**Table 3:** Matrix generated for the CCD, together with the values obtained for each independent variable (±SD) considered for system optimization.

Run	Lipid (X_1_, mg)	Surfactant (X_2_, mg)	Particle Size (Y_1_, nm)	PdI (Y_2_)	Z-Pot (Y_3_, mV)
1	300.0	400.0	209.8 ± 10.7	0.269 ± 0.04	−13.8 ± 0.51
2	300.0	400.0	197.8 ± 2.5	0.216 ± 0.02	−13.4 ± 0.31
3	200.0	200.0	203.9 ± 1.3	0.273 ± 0.04	−16.7 ± 0.42
4	300.0	400.0	231.7 ± 3.2	0.267 ± 0.01	−13.7 ± 0.49
5	300.0	400.0	201.8 ± 1.0	0.272 ± 0.03	−11.9 ± 1.10
6	200.0	600.0	157.5 ± 2.1	0.354 ± 0.02	−10.6 ± 0.80
7	300.0	400.0	227.6 ± 5.4	0.260 ± 0.02	−12.3 ± 0.44
8	300.0	682.8	175.7 ± 1.4	0.177 ± 0.01	−7.0 ± 0.24
9	158.6	400.0	263.6 ± 7.0	0.432 ± 0.01	−8.5 ± 0.20
10	400.0	200.0	438.6 ± 15.9	0.511 ± 0.01	−12.9 ± 0.40
11	400.0	600.0	286.0 ± 1.2	0.320 ± 0.02	−8.0 ± 0.68
12	441.4	400.0	376.3 ± 8.6	0.484 ± 0.04	−11.2 ± 0.40
13	300.0	117.2	642.9 ± 41.9	0.672 ± 0.02	−15.8 ± 0.67

**Table 4:** Main PK parameters obtained for STP and CBD, orally administered from the optimized formulation (NLC STP/CBD) and commercial products.

	STP		CBD	
	NLC STP/CBD	Commercial Product	NLC STP/CBD	Commercial Product
Cmax (mg/L)	12.3	16.7	1.27	0.61
Tmax (h)	2.0	2.0	1.0	0.5
AUC_0–24_ (mg/L/h)	86.2	89.2	8.0	5.4

## Data Availability

The raw data supporting the conclusions of this article will be made available by the authors on request.

## Supplementary Materials

The following supporting information can be downloaded at: https://www.mdpi.com/article/10.3390/pharmaceutics18040503/s1. S1: CICUAL Protocol Approval; S2: CICUAL Protocol Approval Extension; S3: Animals Sanitary Health Certificate.

## Abbreviations

The following abbreviations are used in this manuscript:

AFM	Atomic force microscopy
BP	Benzylparabene
CBD	Cannabidiol
CCD	Central composite design
CI	Crystallinity index
CMC	Critical micellar concentration
DL	Drug loading
DLS	Dynamic light scattering
DS	Dravet syndrome
DSC	Differential scanning calorimetry
EE	Entrapment efficiency
FTIR	Fourier transform infrared spectroscopy
LDE	Laser doppler electrophoresis
HPLC	High-performance liquid chromatography
NLC	Nanostructured lipid carrier
PdI	Polydispersity index
PK	Pharmacokinetic
RSM	Response surface methodology
SEM	Standard error of the mean
SD	Standard deviation
SDS	Sodium dodecyl sulfate
STP	Stiripentol
TGA	Thermogravimetric analysis
TLC	Thin layer chromatography
XRD	X-Ray diffraction
