# Supplementary material for: Lipid Nanoparticles with Stiripentol and Cannabidiol Oil: From Rational Optimization to Preclinical Characterization
Source: Pharmaceutics. 2026 Apr 19;18(4):503. doi: 10.3390/pharmaceutics18040503 (PMC13118642; doi:10.3390/pharmaceutics18040503)
Supplement: Supplementary file 1 [file pharmaceutics-18-00503-s001.zip › S1. CICUAL Protocol Approval.pdf]

cicual
COMITE INSTITUCIONAL DE CUIDADO Y USO DE ANIMALES DE LABORATORIO
Facultad de Ciencias Exactas - UNLP

La Plata, 5 de Julio de 2016

It is hereby certified that the Institutional Animal Care and Use Committee of the Facultad de Ciencias Exactas, Universidad Nacional de La Plata dependence, has reviewed the protocol and procedure for the care and use of laboratory animals entitled "**Rational development of therapeutic nanosystems for the treatment of refractory epilepsy**" and submitted by Luis Bruno-Blanch (Alan Talevi as alternative researcher). This Committee has found that the above procedures are in agreement with local guidelines for vertebrate animal welfare as well as with US Public Health Service and/or European Union policy on this matter (National Research Council, National Academy Press, Washington DC, 2010, and/or European Union Directive for Animal Experiments 2010/63/EU).

The above project is approved, effective on the above date.

Protocol Number 018-06-15

Se deja constancia que el Comité Institucional para el Cuidado y Uso de Animales de Laboratorio (CICUAL) de la Facultad de Ciencias Exactas de la Universidad Nacional de La Plata evaluó el protocolo de utilización de animales presentado por Luis Bruno-Blanch (Alan Talevi como responsable alterno), titulado "**Desarrollo racional de sistemas terapéuticos nanométricospara el tratamiento de la epilepsia refractaria**". Este comité considera que el mencionado protocolo se ajusta a las normas éticas de tratamiento humanitario locales e internacionales que deben aplicarse a los animales vertebrados que se utilizan en investigación biomédica y por tanto lo aprueba en el día de la fecha (National Research Council, National Academy Press, Washington DC, 2010, y/o Directiva de la Unión Europea para Experimentos en Animales, 2010/63/EU).

La aprobación del protocolo será vigente desde la fecha antes indicada y por el lapso de cuatro años.

Número de Protocolo: 018-06-15

Fernando Chiudo

D. HOZBOR

M. LUNDO

PABLO F. PÉREZ

Fernando Chiudo

NATALIA LAUSADA

PROTOCOLO 018-06-15
"Desarrollo racional de sistemas terapéuticos nanométricospara el tratamiento de la epilepsia refractaria"
Responsable: Luis Bruno-Blanch
Responsables Alternos: Alan Talevi
Válido hasta 22 de Junio de 2019
