# Supplementary material for: Lipid Nanoparticles with Stiripentol and Cannabidiol Oil: From Rational Optimization to Preclinical Characterization
Source: Pharmaceutics. 2026 Apr 19;18(4):503. doi: 10.3390/pharmaceutics18040503 (PMC13118642; doi:10.3390/pharmaceutics18040503)
Supplement: Supplementary file 1 [file pharmaceutics-18-00503-s001.zip › S3. Animals Sanitary Health Certificate.pdf]

La Plata, 5 de Noviembre del 2025

CERTIFICADO DE ORIGEN:

Los animales entregados pertenecen a la cepa BALB/cAnNLAE cuyos progenitores proceden del National Institute of Health (USA).

Los animales fueron criados y producidos bajo barreras sanitarias en el LAE - BIOTERIO de la Facultad de Ciencias Veterinarias de la Universidad Nacional de La Plata y se encuentran libres de los microorganismos patógenos especificados en la lista adjunta. Los animales son mantenidos considerando el bienestar de estos bajo un programa de enriquecimiento ambiental.

Fecha de nacimiento: 2/10/25

Habitación Nº: 1

Cantidad: 48

Machos: 48

Hembras:

NOTA: Si se utilizan cajas de transporte del LAE - Bioterio de la FCV – UNLP, los animales enviados han sido preparados bajo condiciones SPF, por lo tanto; al arribo el desembalaje y los procedimientos de transferencia son críticos para el mantenimiento de su estado de salud. Introduzca la/s caja/s de transporte en su establecimiento de acuerdo con sus disposiciones para el ingreso de animales. El Bioterio no se hace responsable por el estado de los animales, una vez que éstos egresan del mismo.

Dr. Miguel Ayala - Director

Dr. Fabricio Maschi
Div. Producción Animal

BIOTERIO CERTIFICADO SEGÚN RESOLUCION SENASA 617/2002 y DISPOSICION ANMAT 9236/2023

Información de contacto:
Calles 60 y 118. C.C. 296.(1900) -LA PLATA - Tel/Fax: (0221) 4211276
e-mail: mayala@fcv.unlp.edu.ar fmaschi@fcv.unlp.edu.ar

Fecha último Control Sanitario: 1/09/2025

Bacterias, Hongos y Mycoplasmas	Resultados Cult	Resultados PCR	Método diagnóstico	
Bordetella pseudohinzzi	Negativo		PCR	
Citrobacter rodentium	0/12	Negativo	Cult	PCR
Clostridium piliforme (Tyzzer)	Negativo		PCR	
Corynebacterium bovis	Negativo		PCR	
Corynebacterium kutscheri	Negativo		PCR	
Klebsiella oxytoca	0/12	Negativo	Cult	PCR
Klebsiella pneumoniae	0/12	Negativo	Cult	PCR
Mycoplasma pulmonis.	Negativo		PCR	
Leptospira spp.	Negativo		PCR	
Listeria monocytogenes	Negativo		PCR	
Proteus mirabilis	0/12		Cult	
Pneumocysitis spp. (P, carinii; P. mutina)	Negativo		PCR	
Salmonella spp.	0/12	Negativo	Cult	PCR
Shigella spp.	0/12	Negativo	Cult	PCR
Staphilococcus aureus	0/12	Negativo	Cult	PCR
Streptococcus pneumoniae	0/12	Negativo	Cult	PCR
Streptococcus Beta hemolítico	0/12		Cult	
Francisella tularensis	Negativo		PCR	
Campylobacter spp.	Negativo		PCR	

Virus	Resultados	Método diagnóstico
Adenovius (MAd-1; MAd-2)	Negativo	PCR
Virus de la Pneumonía (PVM)	Negativo	PCR
Astrovirus del ratón (AstV tipo 1- 2)	Negativo	PCR
Virus Sendai (SeV)	Negativo	PCR
Norovirus murino **(**MNV 1-4)	Negativo	PCR
Virus diminuto del ratón (MVM)	Negativo	PCR
Virus Elevador de la Lactatodeshidrogenasa (LDV)	Negativo	PCR
Virus de la linfocoriomeningitis (LCMV)	Negativo	PCR
Parvovirus del riñón (MKPV)	Positivo	PCR
Parvovirus de la rata NS1(RMV; RPV; H-1; Kilham)	-/-	PCR
Parvovirus del ratón (MPV)	Negativo	PCR
Parvovirus del hámster (HaPV)	-/-	PCR
Rotavirus del ratón (RMV; EDIR)	Negativo	PCR
Hantavirus (Hanta)	Negativo	PCR
Citomegalovirus (CMV)	Negativo	PCR
Virus Tímico del ratón (MTV; MTLV)	Negativo	PCR
Virus Polyoma del ratón (PyV; MptV; K virus)	Negativo	PCR
Virus Theiler Encefalomielitis del ratón (GDVII;FA)	Negativo	PCR

Virus Theiler de la rata (RTV)	-/-	PCR
Reovirus (Reo 1-4)	Negativo	PCR
Virus hepatitis del ratón (MHV1-3; JHM; A59)	Negativo	PCR
Virus de la Sialodacrioadenitis/ Virus Parker (SDAV, RCV-P)	-/-	PCR
Viruela del ratón, Pox virus (Ectromelia)	Negativo	PCR

**Parásitos**	**Resultados** **MO**	**PCR**	**Método diagnóstico**	
Aspiculuris tetraptera	0/12	Negativo	MO	PCR
Entamoeba spp.	0/12		MO	
Eimeria spp.	0/12		MO	
Demodex spp.	0/12		Mo	
Giardia muris	0/12	Negativo	MO	PCR
Criptosporidium spp.	Negativo		PCR	
Hymenolepis spp. (H. diminuta)	0/12	Negativo	MO	PCR
Toxoplasma gondii	Negativo		PCR	
Encephalitozoon spp.	Negativo		PCR	
Myobia musculi	0/12		MO/G/F	
Myocoptes musculinus	0/12		MO/G/F	
Notoedres spp.	0/12		MO/G/F	
Polyplax spp.	0/12		MO/G/F	
Radfordia spp.	0/12		MO/G/F	
Psorergates spp.	0/12		MO/G/F	
Spironucleus muris	0/12	Negativo	MO	PCR
Tritrichomonas muris	0/12		MO	
Syphacia spp. (S. obvelata, S. muris)	0/12	Negativo	MO	PCR
Sarcoptes scabiei	0/12		MO/G/F	

Referencias: Cult (cultivo bacteriológico); PCR se realiza a partir de muestras de ciego, intestino delgado, riñón, pulmón, hígado, ganglios linfáticos mesentéricos y bazo; MO (microscopio óptico); F (flotación) se realiza a partir de un pool de heces; G (test de Graham); -/- no se controla (no es específico en la especie controlada).

Nota: El LAE no se hace responsable por el uso inadecuado de estos resultados

Dr. Juan Martin Laborde

Lic. Martín Carriquiriborde

LAE. Laboratorio de Animales de Experimentación Facultad de Ciencias Veterinarias
Universidad Nacional de La Plata 60 y 118, 1900 La Plata, Argentina
TE 00 54 221 4236663/4236664 Int.440/ TE (directo): 00 54 221 4211276
http://lae.fcv.unlp.edu.ar
